# Accès à l'eau potable et à l'assainissement: cas de la commune d'arrondissement de Douala V (Cameroun)

**DOI:** 10.11604/pamj.2019.33.244.17974

**Published:** 2019-07-23

**Authors:** Dorine Djuissi Tekam, Noel Vogue, Claude Ngwayu Nkfusai, Maurice Ebode Ela, Samuel Nambile Cumber

**Affiliations:** 1 Faculty of Medicine and Pharmaceutical Sciences, University of Dschang, Dschang, Cameroon; 2 Regional Delegation of Public Health Center, Yaoundé, Cameroon; 3 Department of Microbiology and Parasitology, Faculty of Science, University of Buea, Buea, Cameroon; 4 Faculty of Health Sciences, University of the Free State, Bloemfontein, South Africa; 5 School of Health Systems and Public Health, Faculty of Health Sciences, University of Pretoria Private Bag X323, Gezina, 0001, Pretoria, South Africa; 6 Section for Epidemiology and Social Medicine, Department of Public Health, Institute of Medicine, The Sahlgrenska Academy at University of Gothenburg, Gothenburg, Sweden

**Keywords:** Accès, eau potable, assainissement, population Douala V, Access, drinking water, sanitation, population, Douala V^h^

## Abstract

**Introduction:**

L'accès à l'eau potable et à l'assainissement est depuis longtemps l'enjeu d'un combat mené par de nombreux États. Cependant, il représente un combat quotidien pour des centaines de milliers de citadins qui vivent principalement dans les pays en développement. Le gouvernement camerounais avec l'aide des bailleurs de fond ont élaboré des stratégies devant faire de l'assainissement et l'accès à l'eau potable une réalité. Nous avons donc décidé d'évaluer l'assainissement et l'accès à l'eau potable de la sous-division de Douala V.

**Méthodes:**

Il s'est agi d'une étude transversale descriptive. L'étude s'est effectuée sur une période allant de mai en juin 2018. Un échantillonnage aléatoire à deux dégrées était utilisé. Les données ont été collectées à l'aide d'un questionnaire. L'analyse a été faite par épi info version 7.1.3.3.

**Résultats:**

Il ressort de notre étude que 22,47% déversaient ses eaux dans la nature après usage. Puis 65,55% (493/752) de ménages consommaient l'eau de forage. 53,69% de ménage parcouraient entre 1 à 5 km et 49,25% marchaient plus de 15minutes pour avoir de l'eau. 85,50% de ménages n'utilisaient pas une méthode de traitement de l'eau. Seulement 14,49% pratiquaient une méthode de traitement. Aucun ménage n'utilisait la méthode de potabilisation par rayonnement solaire (SODIS). 2/752 ménages (0,26%) n'avaient pas de latrine. La majeure partie des ménages 54,52% (410/752) déversaient les ordures dans la rue.

**Conclusion:**

La mise en place des unités décentralisées: les forages, les bacs à ordures et l'éducation de la population sur les méthodes de traitement de l'eau pour les besoins élémentaires est une urgence.

## Abstract (en)

**Introduction:**

Access to drinking water and sanitation has been a long-standing issue between many States. However, it represents a daily struggle for hundreds of thousands of city dwellers who live mainly in the developing countries. The government of Cameroon with the assistance of providers of funds have implemented strategies to make sanitation and access to safe drinking water a reality. We have therefore decided to assess sanitation and access to drinking water in Douala V sub division.

**Method:**

We conducted a cross-sectional descriptive study from May to June 2018. We used a two-stage random sampling. Data were collected using a questionnaire. The analysis was conducted using Epi Info Version 7.1.3.3.

**Results:**

Our study shows that 22.47% of subjects discharged waters into the natural environment after use. Then, 65,55% (493/752) of households consumed borehole water; 53.69% of households rode between 1 to 5 km, 49.25% walked more than 15 minutes to collect water and 85,50% of households did not use a water treatment method. Only 14.49% of subjects used a water treatment method. No household used solar water disinfection (SODIS); 2/752 households (0.26%) had no latrine. Most of the households (54.52%; 410/752) discharged domestic wastes onto the street.

**Conclusion:**

The creation of decentralized units: the drillings, waste disposal systems and water treatment education to meet basic needs are essential.

## Introduction

L'accès à l'eau potable et à l'assainissement est depuis longtemps l'enjeu d'un combat mené par de nombreux États. Cependant, il représente un combat quotidien pour des centaines de milliers de citadins qui vivent principalement dans les pays en développement [1]. Selon un rapport de l'Organisation Mondiale de la Santé, 319 millions de personnes en Afrique subsaharienne n'ont toujours pas accès à une eau potable soit trois ménages sur quatre vont chercher de l'eau hors de leur domicile et 695 millions de personnes n'ont pas accès à des services d'assainissement de base. Ainsi l'Afrique subsaharienne, l'Afrique du Nord, l'Asie centrale, l'Océanie et la Caucase ont des niveaux de couverture les plus faibles en eau potable [2]. Le nombre de personnes sans accès aux services d'assainissement demeure croissant depuis les années 1990 [2]. Un accès insuffisant à l'eau potable et à un assainissement inadéquat a des graves conséquences sur la santé humaine. Il exacerbe également la pauvreté et freine le développement [3]. C'est la raison pour laquelle la communauté internationale a reconnu depuis 2010 l'accès à une eau de qualité et à des installations sanitaires comme un droit fondamental de l'homme aussi c'est tout l'enjeu des dix-sept (17) Objectifs du Développement Durable (ODD) qui prévoient notamment dans l'objectif 6, un accès universel à l'eau potable et à l'assainissement en 2030. Au Cameroun, le taux d'accès à l'eau potable et aux services d'assainissement est estimé à 3,9% et 34% respectivement [4]. En revanche, selon la Banque Africaine de Développement, le taux de desserte en eau potable était de 33% au Cameroun en 2010 comparé à un pays comme le Sénégal où ce taux était de 98% en zone urbaine et 82% en zone rurale et 67% de Camerounais n'était pas connectés à un réseau d'abduction d'eau [5]. Face à cette situation, le gouvernement camerounais avec l'aide des bailleurs de fond ont mis sur pieds des stratégies d'intervention devant faire de l'assainissement et l'accès a l'eau potable une réalité dans le cadre d'atteinte des Objectifs du Développement Durable (ODD). Préoccupée également par cette situation, la population est obligée de créer eux même des moyens leurs permettant de faire face à ces maux. Mais ceci n'est pas toujours la bien venu aux regards de nombreuses maladies et décès enregistrés toutes les années au sein de la population. Dans ces conditions, des pistes d'intervention doivent être mises en place afin de pallier au mauvais assainissement et au manque d'eau potable. C'est dans cet optique que le projet intitulé « Programme Intercommunal pour la Gestion Durable de l'Eau et de l'Assainissement (PiGeDEA) dans cinq communes du Cameroun notamment la commune de Dschang, Fongo-tongo, Kong-zem, Kyé-Ossi et Douala V a été initié ». Il s'agira de faire un état des lieux actuel de l'assainissement et de l'accès à l'eau potable dans les quartiers de Douala V. Nous nous sommes donc proposé d'évaluer l'assainissement et l'accès à l'eau potable dans l'Arrondissement de Douala V.

## Méthodes

Il s'est agi d'une étude transversale descriptive à base communautaire menée dans les ménages d'Arrondissement de Douala V. L'étude s’est effectuée sur une période allant de mai en juin 2018. La formule statistique Z^2^P (1-P)/d^2^ [6] a été utilisée pour obtenir la taille minimale d'échantillon et 57,4% la prévalence de ménage utilisant une source d'eau de boisson améliorée et une toilette améliorée dans la ville de Douala [7]. La taille minimale de 376 a été déterminée. En tenant compte de l'effet grappe de 2, nous avons obtenu notre taille d'échantillon de 752 ménages. Nous avons mené l'étude dans 30 quartiers sélectionnées de façons aléatoire. Etait éligible pour l'étude tout ménage couvert par la commune d'arrondissement de Douala V et ont été inclus celles ayant consentis à participer à l'étude. Une approbation éthique a été obtenue au Comité d'Éthique Institutionnel de la Recherche pour la Santé Humaine de l'Université de Douala. Une autorisation a été obtenue auprès du maire de la commune de Douala V et un consentement éclaire a été signe par tous les participants consentant de participer à l'étude. Le questionnaire a été prétesté. Les enquêteurs ont été recrutés et formés. L'enquête s'est faite porte à porte et le questionnaire a été administré face à face. Nous avons collecté les informations sur les caractéristiques des ménages et répondants, sur le temps mis pour avoir de l'eau, le nombre de latrine dans les ménages, le mode d'évacuation des ordures et eaux usées, différents mode de traitement de l'eau, la quantité d'eau consomme dans le ménage, les différents points d'eau. Après la collecte, les questionnaires ont été dépouillés et codés. Le masque de saisi des données a été élaboré par EPI INFO version 7.3.1.1 ensuite les données ont été saisies puis analysées.

## Résultats

Le taux de participation était de 100% (752/752). (577/752) 76,72% de ménages étaient majoritairement situés en zone urbaine et 61,03% avaient un sol fait à base de ciment. La moyenne des répondants était de 25 (3,32%) répondants par quartier. La majeure partie des répondants était des femmes avec 538 femmes (71,54%). Les répondants avaient en majorité un niveau d'étude secondaire 415 (59,54%). La moyenne des personnes par ménage était de 6 en zone rurale et 5,9 en zone urbaine (Tableau 1). Concernant le mode d'approvisionnement en eau potable, trois ménages sur quatre utilisaient l'eau de puits pour la vaisselle. Plus de trois ménages sur cinq utilisaient l'eau de forage comme eau de boisson soit 62,73% en zone urbaine et 74,85% en zone rurale. Ainsi, trois ménages sur quartre n'utilisaient aucune méthode de traitement de l'eau et 58,14% se servait d'un filtre comme outil de potabilisation de l'eau. 2/5 ménages qui utilisaient la CDE traitaient de l'eau avant de boire. Ceux qui s'approvisionnaient auprès des forages, puits et sources ne pratiquaient aucune méthode de potabilisation de l'eau à domicile (Tableau 2).

**Tableau 1:** caractéristiques des ménages et cadre de vie

Présentation des Ménages en fonction des zones	Fréquence absolue	Fréquence relative(%)
Zone urbaine	577	76,72
Zone rurale	175	23,27
**Revêtement du sol**		
Ciment	459	61,03
Terre	190	25,26
Carreau	103	13,6
**Réponds en fonction du sexe**		
Féminin	538	71,54
Masculin	214	28,45
**Niveau scolaire**		
Non scolarise	5	0,71
Primaire	217	31,13
Secondaire	415	59,54
Supérieure	60	8,60
**Nombre de personne dans un ménage**		
Zone urbaine	5,9	
Zone rurale	6	
**Nombre d’enfant de moins de 5 ans dans un ménage**		
0	226	30,05
1	334	44,41
2	158	21,01
3	31	4,12
4	3	0,39

**Tableau 2:** mode d’approvisionnement en eau potable

Eau de vaisselle	Fréquence absolue	Fréquence relative (%)
Puits	553	73,53
CDE	186	24,73
Forage	10	1,32
Pluie	3	0,39
**Eau de boisson**		
Forage	493	65,55
CDE	253	33,64
Puits	3	0,39
Pluie	3	0,39
**Traitement de l’eau avant consommation**		
Non	643	85,5O
Oui	109	14,49
**Méthode de traitement N=109**		
Filtre	64	58,71
Chloration	21	19,26
Ébullition	6	5,50
Autres	18	16,27
**Traitement d’eau en fonction des sources**		
**Non**		
CDE		60,40
forage		100
source		100
puits		100
**Oui**		
CDE		39,60

5/6 ménages possédaient au moins une latrine. En zone urbaine 2/577 (0,26%) ménages n'avaient pas de latrine. Par ailleurs, les caniveaux restent un lieu privilégié d’évacuation des eaux usées issues de la lessive, la vaisselle et la cuisine par les ménages tant en zone urbaine (55,80% de ménages) qu'en zone rurale (54,85% de ménages). À peu près deux ménages sur quatre déversent les ordures dans la rue en zone urbaine et trois ménages sur quatre en zone rurale (Tableau 3). À peu près un ménage sur deux de la zone urbaine parcourt entre 1 à 5 km pour avoir de l'eau à boire et plus de deux sur trois en zone rurale. Les populations de Douala V ont une consommation journalière moyenne de 11,34 litres d'eau. Après analyse chaque individu dans un ménage consommait en moyenne 1,89 litre d'eau/jour. Concernant l'accessibilité temporelle, en zone rurale, 49,25% des ménages font plus de 15 minutes de marche (aller et retour) pour avoir de l'eau à boire par contre en zone urbaine seulement 36,80% des ménages le font (Tableau 4).

**Tableau 3:** mode d’accès aux services d’assainissement de base

Ménages ayant au moins une latrine (N: 752)	Fréquence absolue	Fréquence relative (%)
**Oui**		
Zone urbaine	575	99,65
Zone rurale	175	100
**Non**		
Zone urbaine	2	0,34
Zone rurale	00	00
**Mode d’évacuation des eaux usées**		
Caniveaux	418	55,58
Nature	169	22,47
Fosses	144	19,14
Autres	21	2,79
**Évacuation des ordures**		
Rue	410	54,52
Bac a ordure	288	38,29
Autres	54	7,18

**Tableau 4:** accessibilité en terme de distance, temps et quantité moyenne d’eau disponible dans un ménage

Accessibilité en termes de distance	Fréquence relative (%)
Dans le ménage	17,11
Moins d’un km	28,85
Entre 1 et 5 km	53,69
Plus de 5 km	0,35
**Accessibilité en termes de temps**	
**Zone urbaine**	
Moins de 5min	25,97
5 à 10min	21,21
10 à 15 min	16,02
Plus de 15min	36,80
**Zone rurale**	
Moins de 5mins	13,43
5 à10 min	14,93
10 à 15 min	22,39
Plus de 15min	49,25
**Quantité d’eau disponible**	**Quantité (L)**
Zone urbaine	**11,61**
Zone rurale	**10,13**

## Discussion

L'étude que nous avons menée avait pour objectif d'évaluer l'assainissement et l'accès à l'eau potable dans l'Arrondissement de Douala V. D'une part parlant du fait que les répondants sont repartis de manière équitable dans chaque quartier enquêté, ceci pourrait s'expliquer par la méthodologie que nous avons utilisé qui consistait à diviser la taille d'échantillon par le nombre de grappe à enquêter. La majorité (459/752) de ménages ont un sol cimenté, ceci est à peu près semblable à l'étude de [8] dans laquelle 72,5% des ménages avaient un sol fait à base de ciment. Les répondants qui sont constitués majoritairement de femmes que d'hommes s'explique par le fait que le questionnaire était administré aux chefs de ménage et rejoint l'étude menée au Cameroun dans le district de santé de Dschang dans laquelle 60% des répondants étaient des femmes contrairement à celle menée au Benin dans laquelle les répondants étaient à prédominance de sexe masculin (63%) [9,10]. Relatif au niveau scolaire, 55,18% avaient un niveau secondaire ce qui rejoint les résultats de l'étude de Jiokeng où 54,86% avait un niveau secondaire [9]. Par ailleurs ces résultats sont largement différents de l'étude réalisée en Côte-d'Ivoire sur la stratégie d'accès à l'eau potable dans un quartier défavorisé dans lequel 78% des répondants n'ont pas dépassé le cycle primaire. Cette différence peut s'expliquer par le fait que sa recherche s'est réalisée essentiellement dans un quartier précaire de la commune de Cocody en Côte-d'Ivoire lieu d'habitation le plus souvent des analphabètes et des pauvres.

Une taille moyenne de 6 personnes par ménage a été révélée; ce qui n'est pas très diffèrent de l'étude observée en Côte d'Ivoire où la taille moyenne des ménages enquêtés est de 4,7 personnes [8]. Plus de 40% des ménages ont un enfant de moins de 5 ans, résultat semblable à l'étude menée dans les régions de l'Est, de l'Adamaoua, du Nord et de l'Extrême-Nord du Cameroun où 46,5% des ménages enquêtés possédaient un enfant de moins de 5 ans [11]. Concernant la vaisselle, le manque de moyen financier pour se brancher au réseau CDE oblige certains ménages à s'approvisionner auprès des puits. D'où 73,53% des ménages utilisent l'eau de puits. Ceci est conforme aux résultats obtenus dans la commune de Port-Bouet en Côte-d'Ivoire dans laquelle 67% à 75,5% utilisent majoritairement l'eau de puits pour la vaisselle et la lessive [12]. Par ailleurs, l'eau de forage est perçue par les ménages de Douala V comme meilleur source d'eau de boisson ce qui est de nature à pousser certains ménages à leur préférer au détriment des autres points d'eau ceci justifie le fait que 65,58% des ménages l'utilisent comme principale source d'eau de boisson, ce qui rejoint les résultats de l'étude menée dans l'arrondissement de Douala V où la plupart des ménages ont affirmé consommer l'eau de forage comme principale source d'eau à boire [13].

Cependant, une moyenne journalière de 11 litres d'eau potable est disponible pour 6 personnes en moyenne dans un ménage soit 1,8 litre d'eau de boisson disponible ou consommée par personne et par jour; on peut dire que l'accessibilité en terme de volume est acceptable, diffèrent des résultats d'autres études qui montrent qu'une moyenne minimale de 4,5litres d'eau par jour et par personne est nécessaire à un adulte pour maintenir une bonne hydratation [14]. S'agissant du traitement de l'eau, 492 ménages ne traitent pas leur eau avant la consommation dans la zone urbaine pourtant 85 le faisait. Quant à la zone rurale, 151 ménages ne traitaient pas leur eau avant la consommation. Ces effectifs élevés témoignent le fait 66,67% et 83,58% des ménages affirment avoir accès à une eau de bonne qualité en zone urbaine et rurale respectivement. S'ajoute le fait que 100% des ménages s'approvisionnant auprès des forages ne traitent pas leur eau. Par contre 39,60% des ménages qui s'alimentent auprès de la CDE la traitent (58,13% avec un filtre contre 16,27% avec du coton). Nos résultats sont différents des résultats de l'étude menée au Cameroun qui ressort qu'à l'Extrême-Nord et au Nord, utiliser un filtre pour traiter de l'eau est complètement ignoré des ménages. L'ajout de l'eau de javel est le mode de traitement le plus pratiqué par les ménages de toutes les régions sauf celle de l'Extrême-Nord où passer de l'eau dans un linge se positionne au premier rang des modes de traitement d'eau [11]. Cette différence peut s'expliquer par le fait que la majorité des chefs de ménage enquêtée avaient un niveau secondaire. Les populations de Douala V sont soucieuses d'hygiène et d'assainissement ceci se justifie par 99% de ménages ayant au moins une latrine. Ce résultat rejoint le résultat de l'étude menée à Bafoussam qui révèle que le mode d'évacuation des excrétas reste à plus de 95% des latrines à fond perdue [1]. Cependant, 56% des ménages déversent leurs eaux usées dans les caniveaux, les champs et les ravins. L'inefficacité des services de ramassage d'ordure, le fait que certains ménages estiment qu'en saison pluvieuse l'eau doit transporter tous les ordures déversées dans les ravins, une partie importante (54,52%) des ménages déversent leurs ordures dans la nature après les avoir entassées et ramassées à l'aide d'un vieux sceau ou sac poubelle et 14% sollicitent les déversées dans les champs ou ravin résultats semblables à l'étude menée à Bafoussam ou la majeure partie déverse les ordures soit dans les rues, dans les cours d'eau. Comme biais de l'étude nous pouvons avoir des biais d'information qui révèle du fait que certains ménages peuvent avoir menti sur des questions.

## Conclusion

Le problème d'approvisionnement en eau et aux services d'assainissement de base se pose avec acuité dans l'arrondissement de Douala V. La plupart de ménages parcourent plusieurs kilomètres à pieds et met plus de 15 minutes pour avoir de l'eau. On enregistre encore des ménages n'ayant pas de latrine. L'inefficacité des services de ramassages des ordures ménagères et l'absence des fosses protégées pour des eaux usées sont des problèmes rencontrés. Ces informations permettent de faire un état des lieux afin de mieux cibler les actions.

### Etat des connaissances actuelles sur le sujet

- Les différents points d'eau sont insuffisants et très éloignés;
- La présence des services d'assainissement est quasi absente;
- La distance et le temps mis pour avoir de l'eau sont longs; l'utilisation des méthodes de traitement de l'eau est très faible.

### Contribution de notre étude à la connaissance

- L'article propose une voie (les populations ont declaré que l'eau provenant des canalisations de la CDE est de mauvaise qualité et font recours aux forages, puits, et ne traitaient pas leur eau avant de la consommer. Sur ceux il est necessaire de faire l'education et la sensibilisation de la population sur la qualité de l'eau distribuée par la CDE et sur les bienfaits d'une eau potable), pour le guide dans la mise en œuvre de projet visant à lutter contre les maladies diarrhéiques;
- Cette étude servira de document de référence (le faible taux (14,49%) d'utilisation d'une méthode de traitement de l'eau montre que les eaux busées doivent être directement consommé) pour les organismes internationaux, des fonctionnaires de différents pays de promouvoir et soutenir les actions sur le traitement de l'eau à domicile;
- Elle apporte des informations telles que (l'évacuation des excrétas se fait dans des latrines à fond perdue qui côtoient les puits, les sources d'eau contribuant à la contamination de l'eau, l'absence des services de ramassage des ordures, les eaux usées qui sont évacuées dans la nature ou dans des rigoles stagnant dans des concessions: elles témoignes une dégradation continue et permanente de l'environnement) qui permettront de soutenir fermement l'utilisation d'approches rationnelle pour améliorer la couverture en matière d'assainissement au moyen de technologies financièrement abordables, efficaces et écologiques.

## Conflits d’intérêts

Les auteurs ne déclarent aucun conflit d'intérêts.
